# Pheochromocytoma: Clinical Experience From a Single Tertiary Care Center in India

**DOI:** 10.7759/cureus.41671

**Published:** 2023-07-10

**Authors:** Chirag LU, Altaf A Naushad, Manjunath P R, Pramila Kalra, Chitra Selvan, Ganavi Y P, Bharathi Kolla, Sagar Sourabh, Devamsh GN, Nikitha S

**Affiliations:** 1 Endocrinology, Ramaiah Medical College, Bangalore, IND; 2 Gastroenterology, St. Johns Medical College, Bangalore, IND

**Keywords:** hypertensive crisis, hypertension, india, clincal experience, catecholamines, pheochromocytoma, adrenal

## Abstract

Introduction: Pheochromocytoma is a catecholamine-secreting tumor arising from adrenomedullary chromaffin cells that has a varied clinical presentation. Identification of this tumor, which has episodic symptoms, is a diagnostic challenge for clinicians. Diagnosis at an appropriate time is important because it is associated with significant morbidity and mortality. This study aims to mitigate the limited availability of data in our geographical area.

Aims and objectives: To assess the clinical, biochemical, and radiological features and outcomes of patients diagnosed with pheochromocytoma at our center.

Materials and methods: This is a retrospective study. Patients diagnosed with pheochromocytoma during 2015-2023 were included in the study. Clinical, biochemical, and radiological data were collected at presentation, post-surgery, discharge, and until the last follow-up; data were retrieved from hospital records. Statistical analysis was done using IBM Corp. Released 2011. IBM SPSS Statistics for Windows, Version 20.0. Armonk, NY: IBM Corp.

Results: This study included 19 patients, of whom 10 (52.6%) were female. The most common clinical presentation was a hypertensive crisis in patients with pre-existing hypertension (63.1%), followed by headache (47.3%). The classical triad of headache, palpitation, and sweating was seen in only three patients (15.7%). The mean tumor size was 5.01±2.06 cm, with a range of 2.5 to 12 cm. All patients underwent adrenalectomy; six patients (31.5%) had perioperative complications, with post-operative hypotension being the most common at 21% (n = 4), followed by an acute coronary event during alpha blockade in one patient (0.05%) and an intra-operative hypertensive crisis in one patient (5%). A biochemical remission rate post-surgery was achieved in 17 (89.47%) patients.

Conclusions: Hypertensive crisis in patients with pre-existing hypertension was the predominant presenting feature in most of our patients. Female predominance was noted (52.3%) compared to males. Perioperative complications were observed in 31.5% of patients, with post-operative hypotension being the most common complication.

## Introduction

Pheochromocytoma is a rare neuroendocrine tumor that can occur sporadically or as part of hereditary syndromes [1]. The symptoms due to the hypersecretion of catecholamines can mimic more than 30 medical disorders. This rare tumor can be lethal if left undiagnosed. Thus, rapid recognition is vital [2]. Max Schottelius (1849-1919), a pathologist in Freiburg, Germany, was the first person to describe the histopathological features of pheochromocytoma; in 2017, Schottelius was finally profiled. He provided the histologic description of bilateral adrenal tumors in an 18-year-old woman who died in 1886 after a long history of panic attacks, tachycardia, and sweating [3].

The diagnosis of pheochromocytoma in India is often delayed due to a lack of awareness, inadequate diagnostic facilities, and the nonspecific nature of the symptoms associated with the condition [4]. However, with the availability of advanced imaging techniques and biochemical tests, the diagnosis of pheochromocytoma in India is becoming more accurate and timelier.

There are several reports and data available on pheochromocytoma from centers in developed countries, but there is limited information available from developing regions of the world. This study aimed to look into the available demographics, clinical presentation, imaging, and biochemical features of pheochromocytoma and the outcome of these patients.

## Materials and methods

A retrospective cross-sectional study was performed on patients with pheochromocytoma presenting to a tertiary care center between January 2015 and March 2023. The study protocol was approved by the Institutional Review Board.

Clinical information for all patients with histologically proven pheochromocytoma was retrieved from hospital records. For each patient, information at presentation, post-surgery, discharge, and until the last follow-up was retrieved by the investigators and recorded in a predefined questionnaire. Hypertensive crisis was considered a presentation if systolic blood pressure (BP) was more than or equal to 180 mm hg and/or diastolic BP was more than or equal to 120 mm hg with or without end organ damage.

All patients underwent pre- and post-surgery hormonal assessment for pheochromocytoma, and the results were obtained from hospital records and noted in a questionnaire. The laboratory tests included a 24-hour urine vanillylmandelic acid (VMA) test until 2018. After 2018, plasma metanephrine and normetanephrines were measured.

Imaging studies such as computed tomography (CT) scans, magnetic resonance imaging (MRI), iodine I 123-metaiodobenzylguanidine (MIBG) scintigraphy, and gallium 68 donate PET scans were done based on the patient's indication and affordability. Tumor size was determined from histological samples obtained from the patient's medical records. Patients were monitored after surgery using both biochemical and radiological methods at regular intervals. The success of the surgery was evaluated by measuring plasma metanephrine and normetanephrine levels, which were considered normal if cured. Independent variables included age, sex, body mass index, and tumor characteristics such as stage, grade, and histology. The dependent variable was the outcome response, which was either alive or deceased.

The IBM Corp. Released 2011. IBM SPSS Statistics for Windows, Version 20.0. Armonk, NY: IBM Corp. was used for statistical analysis, which included descriptive statistics and calculations of frequencies and percentages for categorical data such as gender. Continuous variables were reported in terms of mean and standard deviation for age.

## Results

A total of 19 patients were included in the study. The mean age at presentation was 52.2 + 11.6 years. The rest of the demographic data and baseline characteristics are given in Table 1.

**Table 1:** Baseline characteristics of patients with pheochromocytoma at presentation (n = 19) BMI: Body mass index, BP: Blood pressure

Characteristic	(N =19)
Mean Age (years)	52.24 + 11.6
Range (years)	27-72
Male	9 (47.36%)
Female	10 (52.63%)
Mean BMI (kg/m2)	25.6±2.58
Diabetes Mellitus (pre-existing)	7 (63.36%)
Diabetes Mellitus (new onset)	4 (36.36%)
Hypertension (pre-existing)	11 (61.11%)
Hypertension (new-onset)	7 (38.8%)
Mean Systolic BP	168± 18.33
Mean Diastolic BP	94 ± 9.72

The most common clinical presentations were hypertension crises in pre-existing hypertension (63.15%), followed by headache (47.3%), and palpitation (47.3%), whereas pheochromocytoma was an incidental finding in six patients (31.5%). The classical triad of headache, palpitation, and sweating was seen in only three patients (15.7%). Only one patient was normotensive. Among the incidentally detected cases, four of them had undergone CT imaging for pneumonia evaluation, and two patients underwent it as part of the workup for abdominal pain. The clinical features at presentation in patients with pheochromocytoma are detailed in Table 2.

**Table 2:** Clinical features at presentation in patients with pheochromocytoma (n = 19)

Clinical Presentation	Total patients (%)
Abdominal Pain	6 (31.5%)
Hypertension Crisis	12 (63.15%)
Headache	9 (47.3%)
Palpitation	9 (47.3%)
Sweating	3 (15.7%)
Incidental	6 (31.5%)
Classical Triad	3 (15.7%)

Plasma metanephrine and normetanephrine levels were used for diagnosis in 11 patients (57.8%), and 24-hour urinary VMA levels were checked in eight patients (42.1%). Seven patients (63.6%) had increased plasma metanephrine and normetanephrine levels. All 11 patients had increased normetanephrine levels. All eight patients who underwent 24-hour urine VMA testing showed elevated values.

At presentation, 17 patients (89.47%) had tumor localization by CT scan and the rest by magnetic resonance imaging (MRI). The Hounsfield units (HU) value on CT was available for 14 patients, with a mean of 26.38 ± 5.2 (HU). Iodine-123-meta-iodobenzylguanidine (MIBG) scans were done in four patients with a tumor size of 5 cm or more to rule out metastases. No patient showed evidence of metastasis preoperatively. Pheochromocytoma of the Adrenal Glands Scaled Score (PASS) was done in five of the 19 patients. One patient had a PASS score of eight, and the rest scored less than four.

All patients underwent preoperative blockade. Phenoxybenzamine was used in 17 (89.47%) and prazosin in two patients (10.5%) as an alpha blockade. The mean doses were 74.2 ±9.6 mg and 20 mg, respectively. Metoprolol extended-release formulation was used after adequate alpha blockade in all of the patients, with a mean dose of 84.2± 12.1 mg.

Open adrenalectomy was performed in 14 (73.68%) patients, whereas five (26.3%) patients underwent laparoscopic adrenalectomy. The tumor characteristics, hormonal levels, and perioperative complications are presented in Tables 3, 4, respectively.

**Table 3:** Table showing salient tumor characteristics

Tumor characteristics	N=19 (%)
Right sided	10 (52.63%)
Left sided	9 (47.36%)
Mean tumor size (cm)	5.01±2.06
Range (cm)	2.5- 12

**Table 4:** Hormonal evaluation of the patients

Hormone (units) (upper limit)	Median (Range)	Normal values
Plasma Metanephrines (pg/ml)	146 (33-387)	(12-65)
Plasma Normetanephrines (pg/ml)	786 (398-7200)	(20-196)
24-Hour Urine Metanephrines (mcg/24 hours)	968 (789-1132)	(75-375)

On histopathology, the mean tumor size was 5.1 cm (2.5 cm to 12 cm). Six patients (31.5%) had perioperative complications. Four patients had post-operative hypotension, which was managed with intravenous fluids, steroids, and vasopressors and recovered with a mean duration of two days (1 to 4 days). One elderly patient who had a pre-existing ischemic heart disease managed medically with an ejection fraction of 58% had an episode of acute decompensated heart failure three days after initiation of the alpha blockade. One patient had an intraoperative hypertensive crisis managed with nitroglycerin and beta-blockers (Table 5).

**Table 5:** Perioperative complications in patients with pheochromocytoma (n =19)

Complications	(N=19) (%)
Post operative hypotension	4 (21.05%)
Acute decompensated heart failure	1 (0.05%)
Intraoperative hypertensive crisis	1 (0.05%)

A complete biochemical and structural cure post-surgery was achieved in 17 (89.47%) patients. Although control of blood pressure was achieved in all patients compared to baseline, nine patients required anti-hypertensives, and six patients required anti-diabetic drugs in due course of time. The mean patient follow-up was 16.5 months (2 months to 58 months). Two of the patients who had persistent hypertension had a hemorrhagic cerebrovascular accident but survived.

## Discussion

The study included 19 participants with a mean age of 52.24 years, ranging from 27 to 72 years. Out of the total participants, nine (47.36%) were male, and 10 (52.63%) were female. In a study by Gupta et al. [5], 71 patients with pheochromocytoma were included, of whom 47 (66.2%) were males and 24 (33.8%) were females. The mean age of presentation was 36.4 years, with a range of 13 to 65 years. The study found that most patients (59.2%) were below the age of 40 at presentation.

A large international study by Lenders et al. [6] investigated the clinical and genetic characteristics of pheochromocytoma and paraganglioma in a cohort of 1,242 patients from 10 different countries. The study found that the mean age of onset was 42.6 years, with a range of 6 to 86 years. The age of onset of pheochromocytoma varies according to different factors, such as genetic mutations and geographical location.

In a study conducted by Pacak and colleagues [7], the authors investigated the clinical and biochemical characteristics of 176 patients with pheochromocytoma. They found that the most common clinical presentations were hypertension (84%), headache (60%), and sweating (59%). The authors also reported that 11% of patients were asymptomatic and were diagnosed incidentally. About 31.5% (six patients) were diagnosed incidentally, and only 15.7% (three patients) presented with the classical triad of symptoms. 

In our study, most of the patients who were previously diagnosed with hypertension presented with hypertensive crises. The classic triad of clinical features, including episodic headaches, palpitations, and diaphoresis, is present in only about 10% of cases.

The use of 24-hour urinary catecholamine metabolites was predominant before 2018, and thereafter plasma metanephrines and normetanephrine were the standards of care at our center after studies showed increased sensitivity and specificity [8]. In a study conducted by Bhatia et al. [9], which included 50 patients with pheochromocytoma, of whom 25 (50%) were males; and 25 (50%) were females. The study found that all patients had elevated levels of urinary catecholamines, with 40 patients (80%) having elevated levels of metanephrines. Plasma-free metanephrines were found to be elevated in 43 patients (86%), while plasma normetanephrines were elevated in 37 patients (74%). In our study, all the patients tested with plasma normetanephrine and 24-hour urine VMA accurately diagnosed Pheochromocytoma; four patients (21%) had normal plasma metanephrine levels, which would have been missed if plasma normetanephrine was not measured concurrently.

The tumor characteristics of 19 patients with pheochromocytoma were analyzed, and the results are presented as follows: 10 patients (52.63%) had tumors located on the right side, while nine patients (47.36%) had tumors located on the left side. The mean tumor size was 5.01±2.06 cm, with a range of 2.5 to 12 cm. In a study by Eisenhower G. et al. [10], which included 225 patients with pheochromocytoma, CT imaging was performed in all patients. The study found that tumor size was significantly associated with symptoms such as headaches, palpitations, and sweating. The study also found that larger tumors (> 6 cm) were more likely to have metastasized than smaller tumors. 

The Hounsfield units (HU) value on CT was available for 14 patients; it ranged from 18 to 58 HU with a mean of 26.38 HU. In the Mayo Clinic cohort [11], the mean unenhanced CT attenuation was 35±9, and only 15 patients (7.5%) had HU less than 20. In our study, two patients (10.5%) had HU less than 20, and none of the patients had HU less than 10, which was in accordance with the conclusion of the study, which stated that biochemical analysis may not be required to rule out pheochromocytoma if an adrenal incidentaloma has a low attenuation (<10 HU). 

The preoperative alpha blockade was done with phenoxybenzamine in most of our patients, and metoprolol was used for beta-blockade. In a study by Karmakar et al. [12], preoperative alpha blockade with phenoxybenzamine was effective in controlling blood pressure in all patients. The study also found that beta blockade with propranolol was effective in reducing heart rate in 12 of 14 patients. The study concluded that preoperative alpha and beta-blockade drugs are effective in controlling blood pressure and heart rate in patients with pheochromocytomas, and they should be used to prevent perioperative complications. In a study by Lenders et al. [13], alpha and beta-blockade drugs were studied in 142 patients with pheochromocytomas. The study found that preoperative alpha blockade with phenoxybenzamine was effective in controlling blood pressure in 95% of patients. The study also found that beta blockade with propranolol was effective in reducing heart rate in 71% of patients. The preoperative alpha blockade was done with phenoxybenzamine in most of our patients, and metoprolol was used for beta-blockade. One elderly patient who had ischemic heart disease being managed medically with aspirin, diuretics, and beta blockers had an acute decompensated heart failure event during day three of the alpha blockade. The possible mechanisms for the cute decompensation could be due to the temporary cessation of diuretics and beta blockers to initiate alpha blockade. One patient had an intraoperative HTN crisis, but the rest of the patients achieved target BP and heart rate.

Surgical resection is the primary treatment for pheochromocytoma, and postoperative monitoring is recommended. A study by Rao et al. [14] compared the outcomes of laparoscopic and open adrenalectomy in 43 patients with pheochromocytomas. The study found that laparoscopic adrenalectomy was associated with less blood loss, a shorter hospital stay, and a faster recovery compared to open adrenalectomy. In a study by Lee et al. [15] on 125 subjects, laparoscopic adrenalectomy was associated with less blood loss, a shorter hospital stay, and a faster recovery compared to open adrenalectomy. Open adrenalectomy was performed in 14 (73.68%) patients, whereas five (26.3%) patients underwent laparoscopic adrenalectomy. In two patients, the initially planned laparoscopic surgery was converted to an open adrenalectomy.

In a study by Desai et al. [16], the correlation between the PASS score and the clinical outcome was studied in 15 patients with pheochromocytomas. The study found that high PASS scores (>8) were significantly associated with malignant behavior and a poor prognosis. In a study by Thompson et al. that assessed the utility of the PASS score in predicting the biological behavior of pheochromocytoma in a large cohort of patients. The study found that high PASS scores (>8) were significantly associated with malignant behavior, recurrence, and poor survival. Pheochromocytoma of the Adrenal Glands Scaled Score (PASS) was done in five of the 19 patients in our study. One patient had a PASS score of eight, and the rest had a score of less than four.

Seventeen patients had biochemical remission when assessed in the postoperative period (89.47%). A study by Singhal et al. [17] evaluated the rate of immediate postoperative remission in 45 patients with pheochromocytoma who underwent surgical resection. The study found that 93.3% of the patients achieved immediate postoperative remission. The study also found that patients with smaller tumors had a higher rate of immediate postoperative remission than those with larger tumors. In a study by Pacak et al. [18], the rate of immediate postoperative remission was assessed in a large cohort of patients with pheochromocytoma. The study found that 88% of the patients achieved immediate postoperative remission. The study also found that patients who received preoperative alpha-adrenergic blockade had a higher rate of immediate postoperative remission than those who did not.

A study by Abraham J. et al. [19] evaluated the follow-up rates in 50 patients with pheochromocytoma who underwent surgical resection. The study found that only 54% of the patients had regular follow-up after surgery. The study also found that patients who had a family history of pheochromocytoma had a higher rate of regular follow-up than those who did not. A study by Hamidi et al. [20] assessed the follow-up rates in a large cohort of patients with pheochromocytoma. The study found that only 60% of the patients had regular follow-ups after surgery. The study also found that younger patients and those with non-metastatic disease had a higher rate of regular follow-up than older patients and those with metastatic disease. Our cohort had a follow-up rate of 42.1%, which reiterates the importance of patient counseling regarding the importance of this disease.

A study conducted in India by Rajan et al. [21] reported perioperative complications in 14% of patients undergoing surgery for pheochromocytoma. The most common complications observed were hypertension (9%) and tachycardia (5%). Other less common complications included arrhythmias, hypotension, and respiratory distress. However, the authors note that all patients in their study were managed successfully, and there were no mortalities reported.

Similarly, a retrospective study conducted in the United States by Wang et al. [22] reported a perioperative complication rate of 23% in patients undergoing surgery for pheochromocytoma. The most common complications observed were hypertension (16.4%) and tachycardia (6.6%). Other less common complications included arrhythmias, hypotension, and pulmonary edema. Among the 19 patients with pheochromocytoma in this study, six (31.58%) experienced perioperative complications. The most common complication was postoperative hypotension, observed in four patients (21.05%). One patient (5.26%) developed NSTEMI during the alpha blockade, and another patient (5.26%) experienced an intraoperative hypertensive crisis.

This study has several limitations, including its retrospective design and reliance on electronic hospital software and patient visits to other clinics within the hospital to extract data, which may introduce recall bias. Additionally, standardized biochemical testing was not performed for all pheochromocytoma patients, and the study was conducted at a single center with a relatively small sample size. Despite these limitations, this study provides valuable insights into the clinical presentation and outcomes of pheochromocytoma in a region where there is limited data available. The favorable cure rate highlights the effectiveness of adrenalectomy as the primary treatment modality. 

## Conclusions

Hypertensive crisis in patients with pre-existing hypertension was the most common clinical presentation, followed by headache and palpitation. Notably, the classical triad of symptoms was observed in a minority of cases. Our study underscores the importance of early recognition and comprehensive evaluation in patients presenting with hypertension crises. Our findings revealed a slightly higher prevalence of female patients and a mean age at presentation of 52.2 years. Postoperative hypotension was the most common perioperative complication in our study. Further research is warranted to explore strategies for long-term blood pressure control and comorbidity management in patients with pheochromocytoma.
